# Intracellular Survival of *Leishmania major* Depends on Uptake and Degradation of Extracellular Matrix Glycosaminoglycans by Macrophages

**DOI:** 10.1371/journal.ppat.1005136

**Published:** 2015-09-03

**Authors:** Thomas Naderer, Joanne Heng, Eleanor C. Saunders, Joachim Kloehn, Thusitha W. Rupasinghe, Tracey J. Brown, Malcolm J. McConville

**Affiliations:** 1 The Department of Biochemistry and Molecular Biology and the Bio21 Institute of Molecular Science and Biotechnology, University of Melbourne, Parkville, Victoria, Australia; 2 Department of Biochemistry and Molecular Biology, Monash University, Clayton, Victoria, Australia; 3 Metabolomics Australia, Bio21 Institute of Molecular Science and Biotechnology, University of Melbourne, Parkville, Victoria, Australia; Institute Pasteur, FRANCE

## Abstract

*Leishmania* parasites replicate within the phagolysosome compartment of mammalian macrophages. Although *Leishmania* depend on sugars as a major carbon source during infections, the nutrient composition of the phagolysosome remains poorly described. To determine the origin of the sugar carbon source in macrophage phagolysosomes, we have generated a N-acetylglucosamine acetyltransferase (GNAT) deficient *Leishmania major* mutant (*∆gnat*) that is auxotrophic for the amino sugar, N-acetylglucosamine (GlcNAc). This mutant was unable to grow or survive in *ex vivo* infected macrophages even when macrophages were cultivated in presence of exogenous GlcNAc. In contrast, the *L*. *major ∆gnat* mutant induced normal skin lesions in mice, suggesting that these parasites have access to GlcNAc in tissue macrophages. Intracellular growth of the mutant in *ex vivo* infected macrophages was restored by supplementation of the macrophage medium with hyaluronan, a GlcNAc-rich extracellular matrix glycosaminoglycan. Hyaluronan is present and constitutively turned-over in *Leishmania*-induced skin lesions and is efficiently internalized into *Leishmania* containing phagolysosomes. These findings suggest that the constitutive internalization and degradation of host glycosaminoglycans by macrophages provides *Leishmania* with essential carbon sources, creating a uniquely favorable niche for these parasites.

## Author Summary

Macrophages are the primary host cells for a number of important microbial pathogens, including protozoan parasites belonging to the genus *Leishmania*. With few exceptions, little is known about the nutrient composition of the vacuolar compartments occupied by these pathogens. *Leishmania* proliferate within the mature phagolysosome compartment of macrophages and recent studies have suggested that intracellular parasite stages are dependent on the uptake of amino sugars. However, how *Leishmania* gain access to these sugars is unclear. In this study we have generated a *Leishmania major* mutant that is a strict auxotroph for the amino sugar, N-acetylglucosamine (GlcNAc). This mutant exhibited a similar virulence phenotype as wild type parasites in infected mice, but was unable to survive in cultured macrophages. The intracellular survival of the GlcNAc-auxotroph in cultured macrophages was restored by supplementation of the medium with the high molecular weight glycosaminoglycan, hyaluronan, which is rich in GlcNAc. Hyaluronan is a major component of vertebrate extracellular matrix and we show that it is rapidly degraded in *Leishmania*-induced skin lesions. Hyaluronan is internalized by infected macrophages and traffics to the *Leishmania* containing phagolysosome. *Leishmania* thus appear to exploit the critical role of macrophages in extracellular matrix turnover to obtain essential sugar carbon sources for growth and virulence.

## Introduction

Protozoan parasites, belonging to the genus *Leishmania*, cause a number of important diseases in humans, that affect over 12 million people worldwide with more than 2 million new infections each year [1]. Clinical disease ranges from self-healing cutaneous lesions to visceral leishmaniasis, which is invariably fatal if left untreated. There is currently no effective vaccine against *Leishmania* and front-line drug treatments are limited by high toxicity, expense, requirement for hospitalization and the emergence of drug resistance [2].

*Leishmania* are transmitted by a sandfly vector that injects infective metacyclic promastigotes into the skin of the mammalian host during a bloodmeal. Promastigotes are phagocytosed by macrophages that are recruited to the site of the sandfly bite, either directly or after passage through neutrophils [3,4]. Parasites internalized by macrophages are delivered to the mature phagolysosome compartment where they differentiate to the non-motile amastigote stage. While the biogenesis of the *Leishmania-*occupied phagolysosome has been intensively studied [5], relatively little is known about the nutrient environment within this compartment or the specific carbon sources utilized by these parasites. As phagocytosis and subsequent lysosomal degradation constitutes one of the major functions of macrophages, it is likely that the nutrient composition of the phagolysosome will vary depending on the physiological state and activity of host macrophage [6]. During early stages of infection, *Leishmania* likely reside in macrophages that are involved in wound repair and tissue remodeling processes [7], while at later stages of infection they appear to replicate in alternatively activated macrophages that display distinct microbicidal responses and metabolism [8,9]. *Leishmania* also reside within other phagocytic host cells that disseminate to lymph nodes and other organs [5]. However, little is known about the metabolic environment that sustains parasite life under these conditions.

Previous studies have shown that *Leishmania* amastigotes are dependent on the up-take of sugars for growth and virulence in the mammalian host [10]. Specifically, targeted deletion of three high affinity hexose transporters in *L*. *mexicana* severely reduces intracellular growth of amastigote in macrophages [11,12]. Similarly, disruption of a *L*. *major* gene encoding the enzyme, glucosamine-6-phosphate deaminase (GND), required for the catabolism of amino sugars, also results in strong attenuation of amastigote growth in both macrophages and in susceptible mice [13]. The dependence of *L*. *major* amastigotes on GND indicated that these stages have access to amino sugars, such as glucosamine (GlcN) or N-acetylglucosamine (GlcNAc) within the phagolysosome compartment. This is consistent with the finding that a *L*. *major* mutant, auxotrophic for all amino sugars, was able to infect mice and establish normal lesions [14]. Amino sugars are present as free sugars in the blood, and are also major components of a number of mammalian glycoconjugates, including glycoproteins (N- and O-glycans/glycosylphatidylinositol anchors), proteoglycans and glycolipids [15]. However, the extent to which *Leishmania* have access to or can utilize host glycoconjugates as a carbon source has not been investigated.

To further investigate the capacity of *Leishmania* to salvage specific amino sugars, we have generated a *L*. *major* mutant that is a strict auxotroph for GlcNAc. This mutant was capable of establishing large skin lesions in susceptible mice, but failed to survive in cultured macrophages. Analysis of the growth phenotype of this mutant in *ex vivo* infected macrophages showed that intracellular growth was not dependent on the uptake of free amino sugars, but rather was rescued by supplementation of infected macrophages with high molecular weight hyaluronan, an abundant GlcNAc-rich polysaccharide component of the extracellular matrix. We propose that uptake and degradation of hyaluronan provides *Leishmania* amastigotes with essential carbon sources, suggesting that the parasite exploits a major function of macrophages in extracellular matrix turnover and remodeling in the skin and other tissues.

## Results

### Characterization of GNAT in *L*. *major*

**Fig 1:**
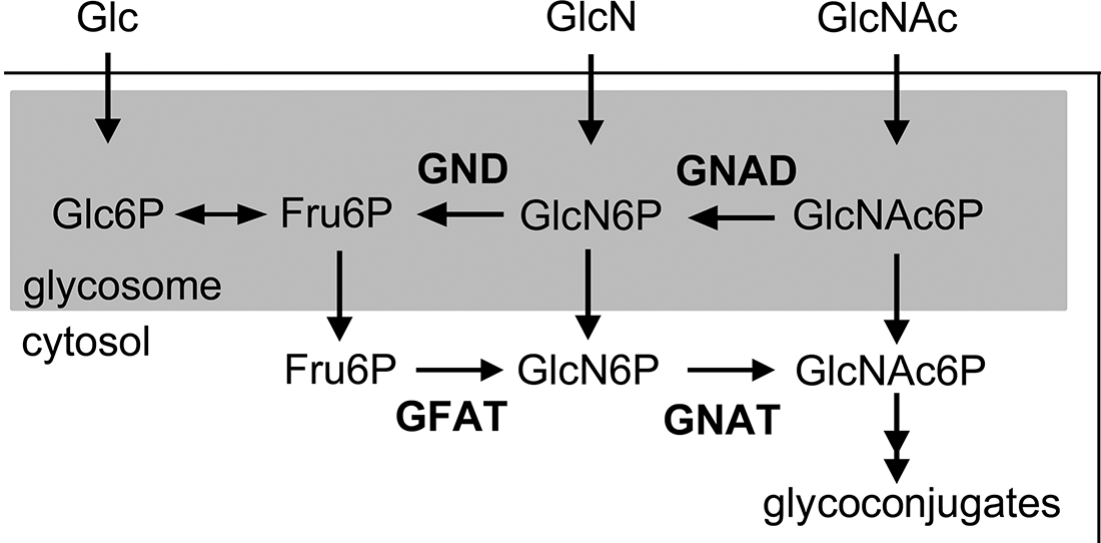
Schematic diagram of hexosamines biosynthesis and catabolism in *Leishmania*. Exogenous sugars, such as glucose (Glc), glucosamine (GlcN) and N-acetylglucosamine (GlcNAc) are taken up via the hexose transporters and phosphorylated within glycosomes by hexokinase. Fructose 6-phosphate (Fru-6P) can be used for *de novo* hexosamine biosynthesis via cytosolic glutamine:fructose-6-phosphate amidotransferase (GFAT) and N-acetylglucosamine acetyltransferase (GNAT), which are essential for the synthesis of glycoconjugates. In contrast, catabolism of GlcNAc-6P depends on the glycosomal glucosamine deaminase (GND) and GlcNAc deacetylase (GNAD), allowing the utilization of GlcNAc as major carbon source.

**Fig 2:**
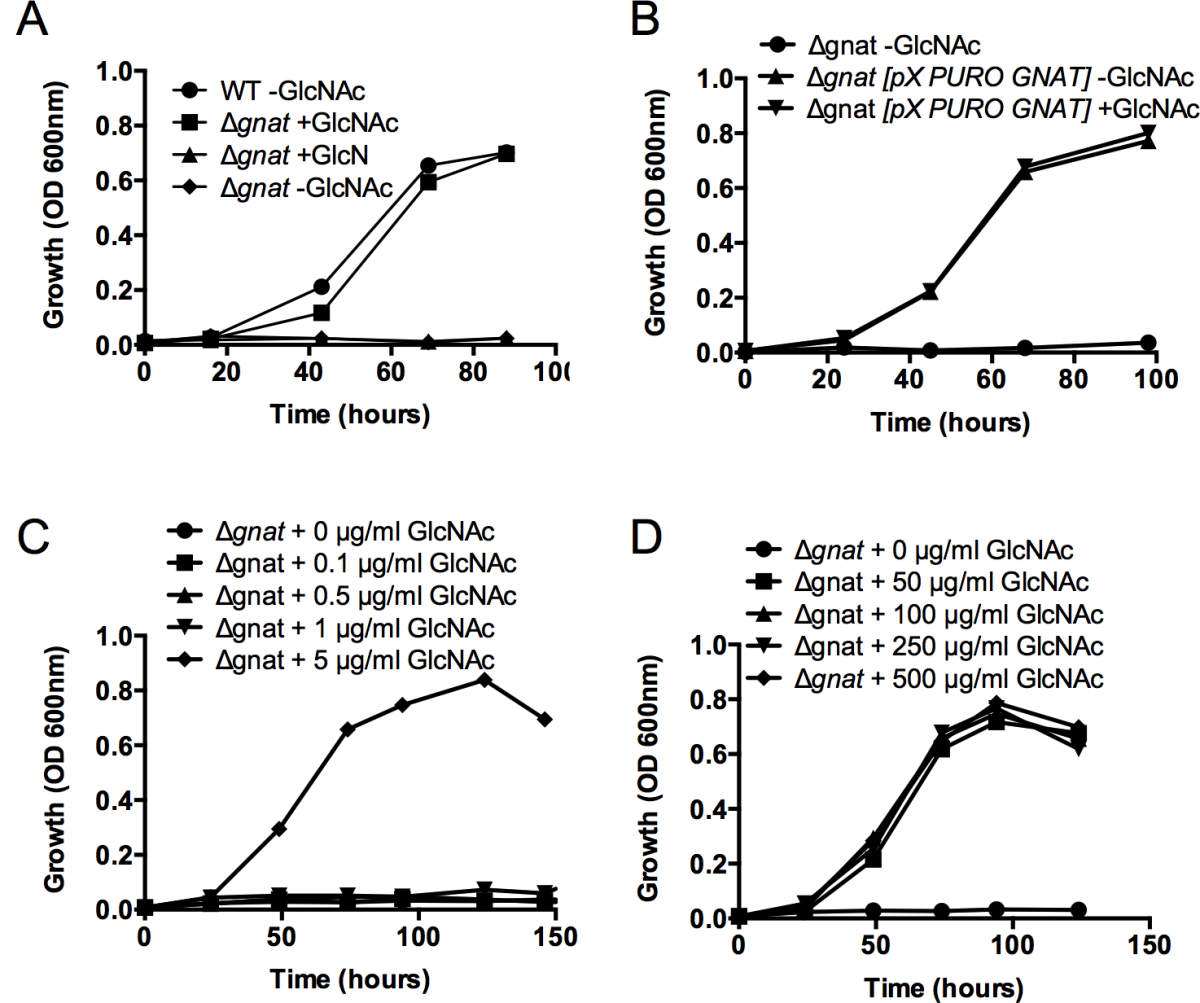
The *L*. *major ∆gnat* mutant is a GlcNAc auxotroph. (A) Wild type (WT) and ∆*gnat* promastigotes were cultivated in M199 medium supplemented with 50 μg/ml GlcN or GlcNAc and parasite growth monitored by measuring optical density at 600nm. (B) ∆*gnat* growth in the absence of GlcNAc was rescued by ectopic expression of GNAT from pXG-PURO plasmid, ∆*gnat* [*pX-PURO-GNAT*], in M199. ∆*gnat* mutant was incubated in (C) limiting or (D) increasing concentrations of GlcNAc and growth monitored over time. Data represents mean from three biological repeats.

*L*. *major* promastigotes can synthesize amino sugar phosphates *de novo* via the hexosamine biosynthesis pathway that includes the enzymes, glutamine:fructose-6-phosphate amidotransferase (GFAT) and GNAT (Fig 1). Targeted deletion of GFAT, the first enzyme in this pathway, results in amino sugar auxotrophy that can be bypassed by supplementation of the medium with either GlcN or GlcNAc (Fig 1) [14]. In contrast, targeted deletion of GNAT, the second enzyme in this pathway, would be expected to lead to amino sugar auxotrophy that could only be bypassed by exogenous GlcNAc, but not GlcN. The *L*. *major* genome contains a gene encoding a putative GNAT (LmjF28.3005) that shares 48% similarity and 28% identity at the amino acid level to the yeast GNAT (ScGNA1p) (S1 Fig). Importantly, amino acids involved in substrate and cofactor binding (Glu98 and Asp99, located in a large hydrophobic cleft) and enzyme catalysis (Tyr143) in the yeast enzyme are conserved in the *L*. *major* GNAT (S1 Fig) [16]. Closely related GNAT homologues are also present in the genomes of the trypanosomatid parasite *T*. *cruzi* (S1 Fig) and have recently been shown to be essential for blood stage *T*. *brucei* [17]. The targeted deletion of the *L*. *major* GNAT was achieved by sequential replacement of the two chromosomal alleles with *SAT* and *BLEO* resistance cassettes by double homologous recombination (S2A Fig). Clones lacking the *GNAT* gene were readily isolated when parasites were cultivated in rich medium containing GlcNAc and loss of the *GNAT* gene confirmed by PCR analysis (S2B Fig). As expected, the growth of the *∆gnat* mutant was dependent on the presence of exogenous GlcNAc (Fig 2A) and complete loss of viability was observed after 72 hours in the absence of GlcNAc (S2C Fig). In contrast, GlcN, which is readily taken up by promastigotes and converted to GlcN6P (the substrate for GNAT) [14] was unable to rescue growth (Fig 2A). Importantly, GlcNAc prototrophy in the mutant was restored by ectopic expression of GNAT from the pXG-PURO plasmid (Fig 2B).

### GlcNAc dependent growth of ∆*gnat* promastigotes

To assess the minimum amount of GlcNAc required for growth, *L*. *major* ∆*gnat* promastigote growth was determined in media supplemented with decreasing concentration of GlcNAc (0–5 μg/ml) over time. *L*. *major* ∆*gnat* parasites displayed normal growth kinetics when medium was supplemented with 5 μg/ml of GlcNAc, while no growth was observed when parasites were supplemented with 1 μg/ml GlcNAc (Fig 2C). Addition of excess sugar to specific *Leishmania* sugar auxotrophs can lead to toxicity as a result of the hyper-accumulation of the cognate sugar phosphate and depletion of ATP [18]. However, addition of 50–500 μg/ml GlcNAc to the growth media had no detrimental impact on parasite growth (Fig 2D), indicating that high GlcNAc levels are not toxic to *L*. *major* ∆*gnat* promastigotes.

### GNAT activity in *L*. *major* promastigotes

**Fig 3:**
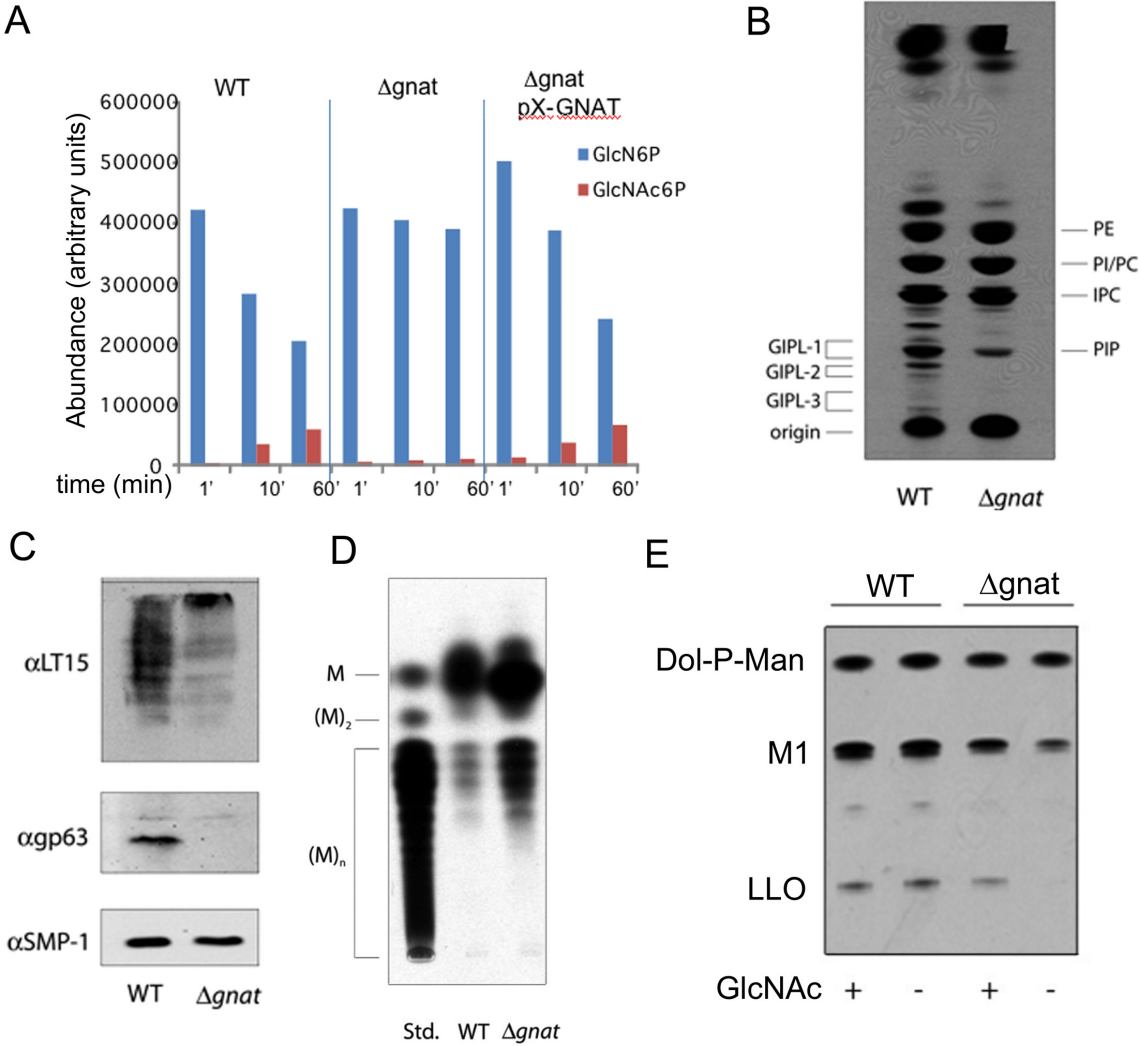
∆*gnat* parasites lack GNAT activity and are defective in glycoconjugate biosynthesis. (A) Cytosolic extracts of wild type (WT), ∆*gnat* and ∆*gnat* [*pX-PURO-GNAT*] cell lines were incubated with 1 mM GlcN6P for indicated times at 27°C. The relative abundances of GlcN6P and GlcNAc6P were determined by direct infusion-mass spectrometry in negative ion mode. (B) WT and Δ*gnat* promastigotes were cultivated in hexosamine-free M199 medium for 24 hours and then pulse labelled with ^3^H-Glc for 30 min. Parasite extracts containing total cellular lipids were analysed by HPTLC and the major phospholipids phosphatidylethanolamine (PE), phosphatidylinositol (PI), inositolphosphatidylcholine (IPC), phosphatidylcholine (PC), phosphatidylinositolphosphate (PIP) and glycolipids (GIPL1, 2 and 3). (C) LPG and gp63 were detected by Western blotting using anti-phosphoglycan repeat antibody, LT15 (top panel) or anti-gp63 (middle panel) antibodies. The flagellar protein, SMP1, was used as a loading control (bottom panel). (D) The neutral glycan mannan contains increasing levels of mannose (M) and was resolved by HPTLC. (E) WT or ∆*gnat* parasites cell lysates were labeled with GDP-[^3^H]Man and dolichol-P-mannose (Dol-P-Man), the GIPL precursor, M1, and a lipid-linked oligosaccharide (LLO) precursors detected by HPTLC.

To confirm that deletion of *L*. *major GNAT* results in loss of GNAT activity, cell lysates of wild type, *∆gnat* and complemented parasite lines were incubated with GlcN6P and the biosynthesis of GlcNAc6P assessed by direct infusion electro-spray ionization mass spectrometry. Synthesis of GlcNAc6P, with a concomitant decrease in GlcN6P levels, was detected in lysates of wild type and complemented parasite lines, but not in lysates generated from the *∆gnat* mutant (Fig 3A). The single *L*. *major GNAT* gene therefore appears to account for all of the GNAT activity.

As GlcNAc is a core component of free GPI and GPI-anchor glycolipids, the synthesis of these glycoconjugates in the ∆*gnat* mutant should be dependent on the presence of exogenous GlcNAc. Indeed, when ∆*gnat* promastigotes were labeled with ^3^H-glucose in the absence of exogenous GlcNAc, the synthesis of free GPIs, but not phospholipids, was completely abrogated (Fig 3B). The GlcNAc-starved ∆*gnat* mutant was also deficient in the expression of the major surface glycoconjugates, LPG and gp63, which are both anchored to the plasma membrane via GPI glycolipids (Fig 3C). As expected, the synthesis of the major intracellular reserve carbohydrate, mannogen, was not abrogated in *∆gnat* promastigotes following removal of GlcNAc (Fig 3D). Rather, increased mannogen levels were observed under these conditions (Fig 3D), which may reflect the diversion of excess hexose phosphates into mannogen synthesis in the absence of parasite growth.

GlcNAc is also a core component of N-linked glycans that are assembled in the ER as lipid-linked oligosaccharides (LLO). The cellular levels of LLO precursor pools can be assessed by incubating *Leishmania* lysates with GDP-^3^H-Man, which results in the rapid labeling of preexisting LLO precursors. As shown in Fig 3E, removal of GlcNAc from the culture medium had no effect on the LLO pool size in wild type parasites, but resulted in the rapid depletion of LLO precursors in the *∆gnat* mutant. Collectively, these results demonstrate that GNAT is required for the synthesis of essential glycoconjugates in the absence of exogenous GlcNAc.

### ∆*gnat* parasites retain virulence in susceptible mice

**Fig 4:**
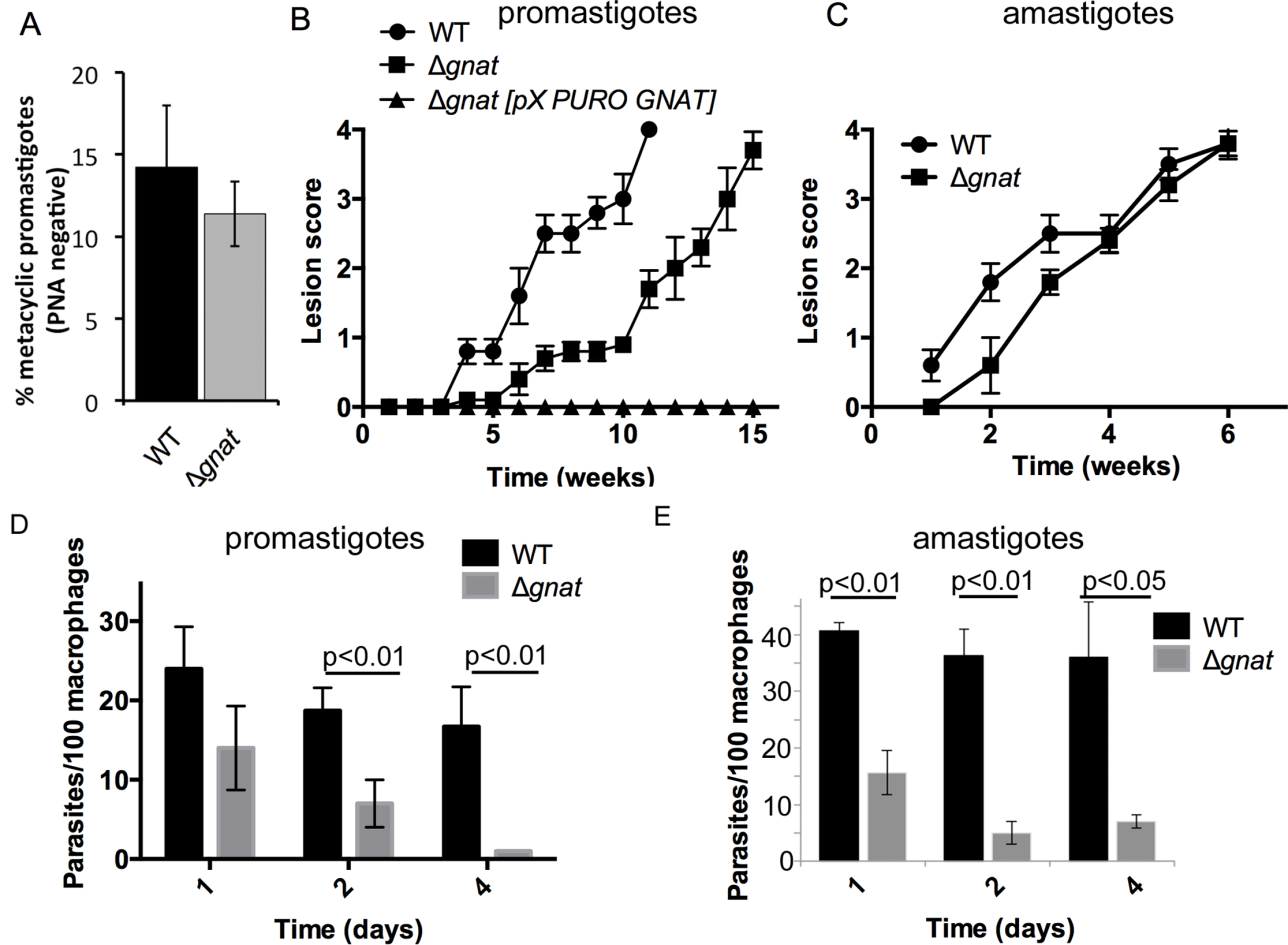
Infectivity of the ∆*gnat* mutant in BALB/c mice and macrophages. (A) WT and ∆*gnat* stationary phase promastigotes were incubated with peanut agglutinin (PNA) and the number of metacyclic (PNA negative) parasites determined. Error bars are SD from three biological repeats. (B) BALB/c mice were infected intradermally with promastigote stages (10^6^ stationary phase) of *L*. *major* wild type (WT), ∆*gnat* and the complemented *∆gnat [pX-PURO-GNAT]* parasite lines. Lesion formation and progression were monitored weekly. Error bars represent standard error of the mean (SEM), n = 5 mice. 1 of 2 independent experiments shown. (C) BALB/c mice were infected intradermally with lesion-derived WT and ∆*gnat* amastigotes (10^6^) and lesion progression was scored weekly. Error bars represent SEM, n = 5 mice. (D) BALB/c bone marrow-derived macrophages were infected with stationary phase *L*. *major* WT and ∆*gnat* promastigotes. Intracellular parasite numbers at days 1, 2 and 4 post-infection were determined from three biological repeat experiments (more than 300 macrophages scored per experiment, error bars represent SEM and p-values are derived from the student’s t-test, two-tailored, unpaired). (E) RAW 264.7 macrophages were infected with lesion derived WT and ∆*gnat* amastigotes and number of intracellular parasites counted over time (mean and SEM from two biological repeat experiments, p-value was calculated by the student’s t-test).

The virulence of *∆gnat* parasites was assessed in the highly susceptible BALB/c mouse model of infection. Infections were initiated with stationary phase wild type and ∆*gnat* promastigotes cultivated in the presence of GlcNAc, which contained similar levels of metacyclic parasites (Fig 4A). Wild type parasites induced lesions within 3–4 weeks, which increased in size over time (Fig 4B). ∆*gnat* parasites also induced large lesions, comparable in size and severity to wild type parasites. However, lesion development was reproducibly delayed by 4–5 weeks (Fig 4B). This delay was also observed with ∆*gnat* promastigotes differentiated from mature lesions, suggesting that it is not a consequence of culture-induced loss of virulence. Unexpectedly, while complementation of the ∆*gnat* mutant reversed the mutant phenotype *in vitro*, it resulted in complete loss of virulence in mice (Fig 4B), raising the possibility that expression of GNAT under non-native conditions is deleterious *in vivo*. In contrast to promastigotes stages, isolated ∆*gnat* amastigotes re-established lesions in mice without a lag phase and comparable kinetics to wild type parasites (Fig 4C). The ∆*gnat* parasites were able to salvage sufficient levels of GlcNAc from the host, as lesion-derived amastigotes expressed free GPIs (GIPL-1, 2 and 3), although at reduced levels compared to wild type amastigotes (S3A Fig). GPI biosynthesis in these amastigotes was not due to restoration of GlcNAc synthesis as isolated ∆*gnat* parasites lacked the *GNAT* gene, as determined by PCR (S3B Fig) and were unable to grow as promastigotes in the absence of exogenous GlcNAc (S3C Fig). These results suggest that while phagolysosomes harboring promastigotes may have limiting levels of GlcNAc, they contain sufficient levels of this amino sugar to promote ∆*gnat* amastigote differentiation and growth and skin lesion formation.

### Growth of *L*. *major* ∆*gnat* promastigotes in *ex vivo* macrophages is dependent on the uptake of exogenous GlcNAc-containing glycosaminoglycans

**Fig 5:**
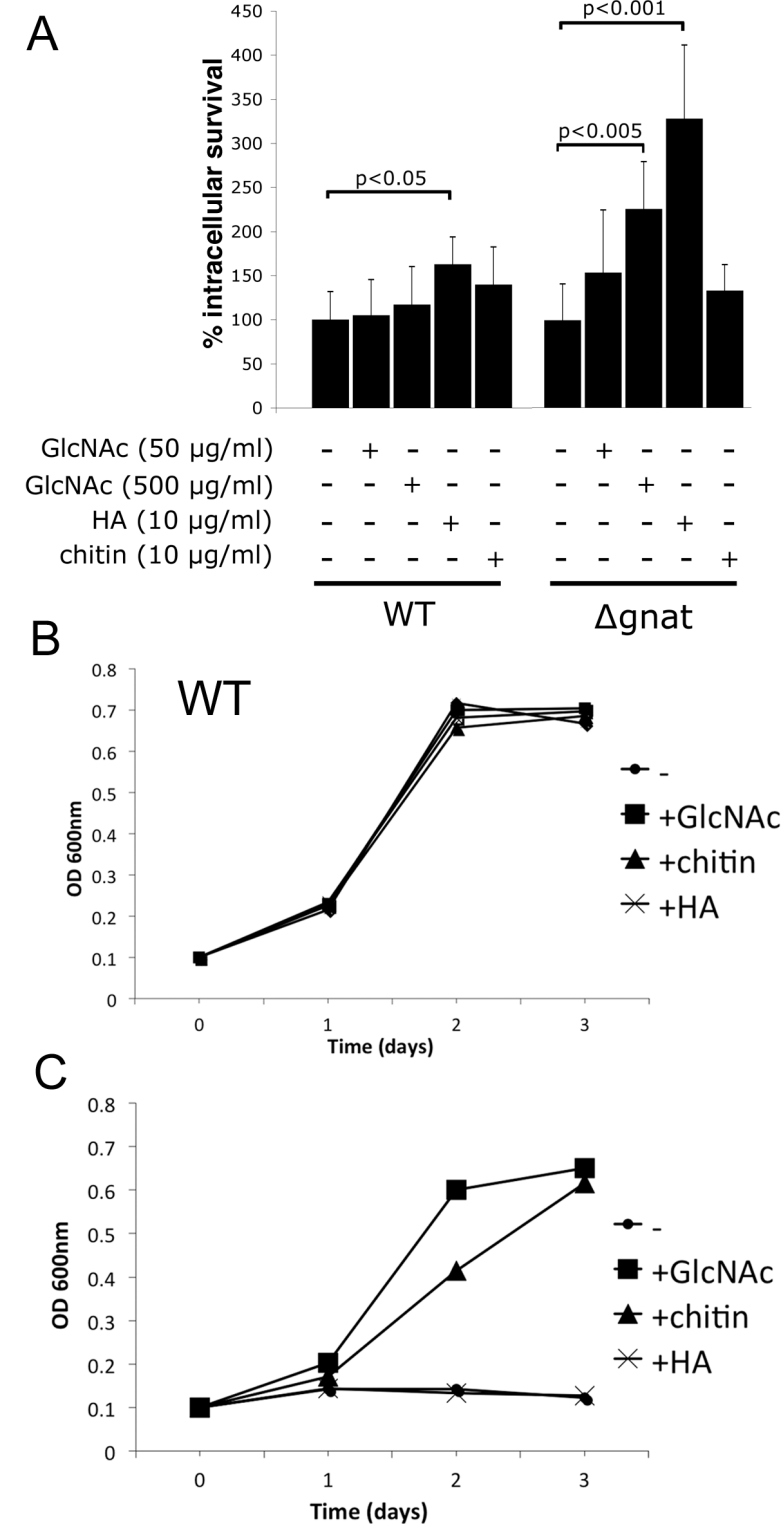
Intracellular ∆*gnat* parasites are rescued by exogenous GlcNAc sources. (A) RAW 264.7 macrophages were infected with wild type (WT) and *∆gnat* stationary promastigotes in the presence of either GlcNAc, hyaluronic acid (HA) or chitin. Intracellular growth was determined after fixation and staining with the DNA dye, Hoechst, and is expressed relative to parasite numbers at day four of untreated cells (whereby WT contained 67 +/- 8 parasites and *∆gnat* 26 +/- 4 parasites). Error bars are the SD from three biological repeat experiments and p-values were calculated by the Student’s t-test. (B) WT and (C) *∆gnat* promastigotes were cultured in the presence of GlcNAc, HA or chitin as the sole hexose source and growth was determined by OD_600_.

To further investigate potential sources of GlcNAc utilized by intracellular amastigotes, infection experiments were undertaken in BALB/c bone marrow-derived macrophages (BMDMs). ∆*gnat* promastigotes were rapidly cleared by BMDMs within four days post infection. In contrast, wild type parasite levels remained constant over the same period (Fig 4D). Similar to promastigotes, lesion derived ∆*gnat* amastigotes were unable to survive in cultured macrophages (Fig 4E), suggesting that *ex vivo* macrophages, but not macrophages in skin lesions, fail to provide sufficient levels of GlcNAc to support intracellular ∆*gnat* growth. To investigate whether intracellular survival of ∆*gnat* parasites could be rescued by exogenous GlcNAc, the medium of infected macrophages was supplemented with 50 μg/ml or 500 μg/ml GlcNAc. Addition of physiologically relevant concentrations of GlcNAc (~40 μg/ml in serum [15]) did not rescue growth of the *∆gnat* mutant, while addition of a 10-fold higher concentration GlcNAc resulted in a modest increase (2-fold) in intracellular growth (Fig 5A). These results suggest that the fluid phase uptake of exogenous GlcNAc and/or transport of GlcNAc from the macrophage cytoplasm to the phagolysosome contribute minimally to the observed *∆gnat* growth in skin lesions.

The extracellular matrix of the dermis contains several glycosaminoglycans, including chondroitin sulfate, heparin and hyaluronan. Hyaluronan is the most abundant of these and the only glycosaminoglycan to contain unmodified GlcNAc within the repeat disaccharide units (GlcAβ1-3GlcNAcβ1–4)_n_, of the polysaccharide backbone. As previous studies have shown that hyaluronan is constitutively turned-over by macrophages in the skin [19], we investigated whether exogenous hyaluronan can rescue the intracellular growth phenotype of growth of *∆gnat* parasites. Addition of high molecular weight hyaluronan to ∆*gnat*-infected macrophages greatly enhanced intracellular survival of *∆gnat* over and above supplementation of infected macrophages with free GlcNAc (Fig 5A). Addition of hyaluronan also resulted in a small but significant stimulation of intracellular growth of wild type parasites (Fig 5A). In contrast, addition of chitin, a polysaccharide containing exclusively GlcNAc, did not increase intracellular ∆*gnat* survival (Fig 5A). To investigate whether the different growth stimulatory properties of hyaluronan and chitin reflected differences in the capacity of the parasites to degrade these polymers, wild type and ∆*gnat* promastigotes were cultured in medium supplemented with GlcNAc, chitin or hyaluronan. Addition of these supplements had no growth inhibitory effect on the growth of wild type parasites (Fig 5B). However, chitin but not hyaluronan had a strong stimulatory effect on ∆*gnat* growth *in vitro*, in contrast to the situation in *ex vivo* infected macrophages (Fig 5C). The capacity of *L*. *major* parasites to utilize chitin is consistent with previous reports showing that promastigotes secrete a soluble chitinase [20,21]. Together, these results suggest that *Leishmania* are unable to degrade high molecular weight hyaluronan directly, but salvage free GlcNAc generated by phagolysosome hyaluronidases and other endo/exoglycosidases.

### Parasite growth is stimulated by high molecular weight hyaluronan polymers

**Fig 6:**
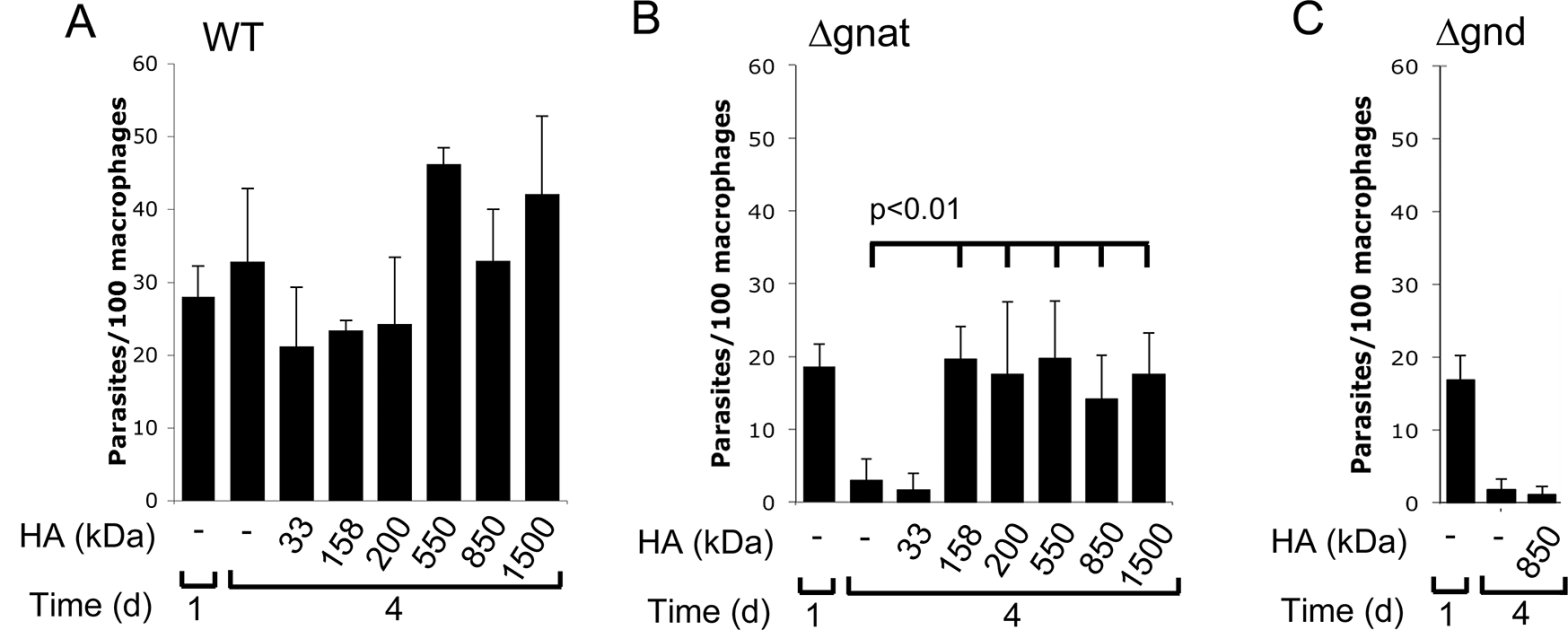
High molecular weight hyaluronan rescue intracellular ∆*gnat* parasites. RAW 264.7 macrophages were infected with (A) wild type (WT), (B) *∆gnat* and (C) *∆gnd* promastigotes in the absence of presence of different molecular weight HA. Intracellular parasites numbers at day 1 and 4 post-infection were determined by fluorescence microscopy after staining with Hoechst. Data represents mean and SD from three biological repeats and p-values were determined by the Student’s t-test.

Hyaluronan is present in the extracellular matrix as high molecular weight polymers (10^3^−10^4^ kDa) which are largely anti-inflammatory. However, lower molecular weight polymers (less than 50 kDa), which can be generated by extracellular hyaluronidases, induce expression of inflammatory cytokines in macrophages [22]. To exclude the possibility that intracellular ∆*gnat* rescue by hyaluronan was due to alterations in macrophage activation state, *∆gnat*-infected macrophages were incubated with a range of hyaluronan polymers with defined molecular weights from 33 to 1500 kDa. Low molecular weight hyaluronan fractions (modal molecular weight of 33 kDa) failed to rescue intracellular *∆gnat* parasites, while high molecular weight hyaluronan fractions (from 158 to 1500 kDa) were highly growth stimulatory (Fig 6B). Growth of wild type parasite was also stimulated by high molecular weight hyaluronan polymers (>500 kDa), although this result did not reach statistical significance (Fig 6A). To further exclude the possibility that the growth promoting properties of hyaluronan was due to an indirect effect on macrophage activation, we tested the *L*. *major* ∆*gnd* mutant which lacks a key enzyme in GlcNAc catabolism [13]. As shown previously, this mutant is unable to replicate in macrophages, providing direct evidence that intracellular amastigote stages are dependent on GlcN or GlcNAc as a carbon source [13]. Strikingly, and in contrast to the ∆*gnat* mutant, intracellular growth of the ∆*gnd* mutant was not rescued by supplementation of the medium of infected macrophages with high molecular weight hyaluronan (Fig 6C). These data suggest that hyaluronan internalization and degradation promotes intracellular parasite growth by providing an essential carbon source.

### Hyaluronan is turned over in skin lesions and traffics to *Leishmania* harboring phagolysosomes

The mammalian skin contains 50% of total body hyaluronan, which is turned over via endocytosis and degradation in lysosomes within 1–2 days [19,23]. To confirm and directly measure hyaluronan turnover in *Leishmania* skin lesions, we measured hyaluronan dynamics in animal tissues using ^2^H_2_O labeling [24]. Infected BALB/c mice were labeled with ^2^H_2_O in their drinking water over several days and the rate of hyaluronan turnover quantitated by measurement of the level of deuterium enrichment in the diagnostic tetrasaccharide repeat unit ([GlcAβ1-3GlcNAcβ1–4]_2_), after extraction, hyaluronidase depolymerization and analysis of the released oligosaccharide fragments by liquid chromatography-mass spectrometry (LC-MS). Deuterium enrichment in the hyaluronan repeat unit increased rapidly during the first five days of labeling, reaching close to maximum labeling observed after four weeks labeling (S4 Fig). These results suggest that hyaluronan is constitutively and rapidly turned over in *Leishmania* lesions with a half-life of <2 days (S4 Fig), consistent with previous reports using healthy skin [23].

**Fig 7:**
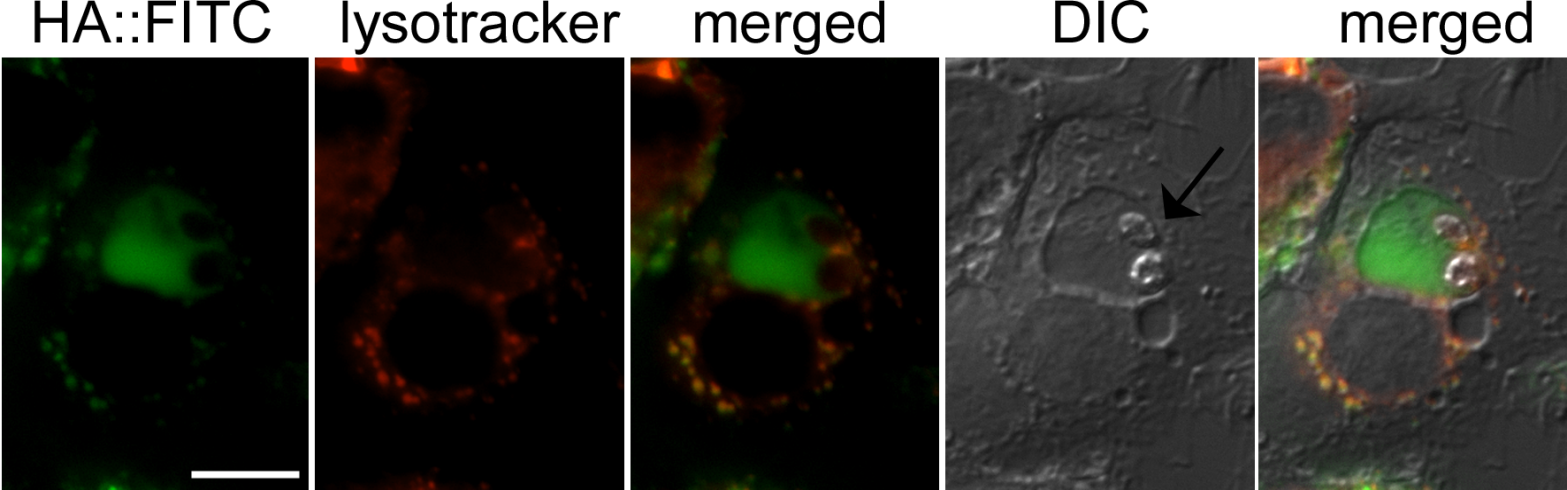
Hyaluronan localization to the *Leishmania* containing phagolysosome. RAW 264.7 macrophages were infected with *L*. *mexicana* promastigotes and then labelled with FITC conjugated hyaluronan (HA::FITC, 10μg/ml) for 24 hours. Macrophage lysosomes were stained with LysoTracker. Live-macrophages were analysed by fluorescence microscopy. Arrow indicates two *L*. *mexicana* amastigotes. Scale bar = 10μm.

To confirm that hyaluronan is indeed internalized into *Leishmania*-containing vacuoles, infected macrophages were incubated with fluorescein-labeled hyaluronan (HA::FITC). When macrophages were infected with *L*. *major* promastigotes expressing mCherry red fluorescent protein, green fluorescence was observed around the periphery of intracellular amastigotes that induce individual, tight fitting vacuoles (S5A Fig). Green fluorescence was readily detected in vacuoles in cases where *L*. *major* induced more spacious compartments (S5B Fig). Similar experiments were performed with macrophages infected with *L*. *mexicana* promastigotes that induce large communal phagolysosomes. HA::FITC accumulated in lysosomal compartments, as evidenced by co-localization with lysotracker, and the *L*. *mexicana* containing phagolysosome (Fig 7), demonstrating efficient endocytosis and lysosomal targeting of these polysaccharides in infected macrophages.

## Discussion

*Leishmania* parasites are dependent on the uptake of sugars for intracellular growth in the mammalian host [12,25]. However, the source of the sugars accessed by intracellular amastigotes has not been defined. We have previously shown that the *L*. *major* mutant lacking GND is unable to catabolize amino sugars as major carbon source and is heavily attenuated in mice infections [13]. In this study we have generated a new *L*. *major* sugar auxotroph that is specifically dependent on salvage of GlcNAc for growth and viability. Using this mutant as a probe for intracellular GlcNAc levels, we show that the levels of this sugar are limiting for parasite growth during early stages of infection, but not for amastigote growth in lesion macrophages. In *ex vivo* infected macrophages, intracellular *∆gnat* amastigotes growth is efficiently restored by supplementation of infected macrophages with the GlcNAc-rich glycosaminoglycan, hyaluronan, but not by supplementation of the medium with physiological concentrations of GlcNAc. Importantly, we demonstrate that *Leishmania* induced skin lesions contain high levels of hyaluronan and that this glycosaminoglycan is constitutively turned over *in situ*. We therefore propose that the constitutive uptake and degradation of hyaluronan by macrophages may partly underlie the tropism for and capacity of *Leishmania* to proliferate within these host cells.

We have previously shown that targeted deletion of *L*. *major* GFAT, the first enzyme in the hexosamine-phosphate biosynthetic pathway, had no measurable effect on amastigote growth and lesion development in mice [14]. The growth phenotype of ∆*gfat* in culture could be by-passed by the provision of either GlcN or GlcNAc, suggesting that either one or both sugars are present within the phagolysosome compartment of macrophages at sufficient levels to sustain amastigote growth at all stages of infection [14]. To further define the amino sugar composition of macrophage phagolysosomes and potential sources of these sugars, we generated the *L*. *major ∆gnat* mutant, which exhibits a restricted auxotrophy for GlcNAc alone. The *L*. *major ∆gnat* promastigotes showed a reproducible delay in skin lesion development, which was also observed with ∆*gnat* amastigotes at a low inoculum (S6 Fig). Nevertheless, *∆gnat* promastigotes and amastigotes induced skin lesions comparable to those induced by wild type parasites. These findings suggest that GlcNAc levels in infected macrophages are limiting during early stages of infection, but are sufficient to sustain normal parasite growth in mature lesions. It is possible that intracellular levels of GlcNAc in infected macrophages increase as lesions develop, possibly as a result of increased turnover of proteoglycans. Alternatively, lesion amastigotes may have a lower requirement for GlcNAc and/or other sugars than amastigotes during early stages of infection. In this respect, we have recently shown that *Leishmania* amastigotes switch to a metabolically quiescent state in lesions, which is characterized by markedly reduced rates of hexose uptake [24]. Taken together, the virulence phenotypes of different *Leishmania* hexose auxotrophs demonstrate that the mature phagolysosomes of macrophages contain sufficient levels of the amino sugar GlcNAc to sustain parasite glycoconjugate biosynthesis and carbon metabolism.

Studies on the intracellular growth of *L*. *major* wild type and mutant lines lacking enzymes in GlcNAc synthesis or catabolism in *ex vivo* cultured macrophages provided direct evidence that amino sugars generated by breakdown of internalized hyaluronan and other proteoglycans are utilized as carbon sources by amastigotes. First, intracellular survival of *L*. *major ∆gnat* promastigotes in macrophages was strongly enhanced by supplementation of the cultures with high molecular weight hyaluronan. In contrast, neither free GlcNAc nor the GlcNAc-rich polymer, chitin, which is mainly degraded extracellularly by macrophages, restored growth. Second, the growth stimulatory activity of high molecular weight hyaluronan was dependent on the expression of the GlcNAc-catabolic pathway in the parasite, as hyaluronan did not rescue the growth defect of the *L*. *major* ∆*gnd* mutant. Third, low molecular weight hyaluronan (<33 kDa), which act as signaling molecule in inflammation, was less effective at rescuing the intracellular growth of *L*. *major ∆gnat* parasites than high molecular weight hyaluronan. The effect of hyaluronan on macrophage signaling and *Leishmania* survival is likely to be negligible, as intracellular survival of wild type *L*. *major* was not affected by low molecular weight hyaluronan. Finally, we show that hyaluronan is internalized into the phagolysosome compartment of cultured macrophages and is actively turned over within macrophage-rich skin lesions with a half-life of < 2 days, comparable to previously determined rates of hyaluronan turnover in healthy skin [19,23]. Our data suggest that the nutrient environment in cultured macrophages may differ substantively from comparable intracellular niches in lesions. In particular, differences in the extent to which *in vitro* cultivated and lesion macrophages take up hyaluronan likely accounts for the modest loss of virulence phenotype of the *L*. *major ∆gfat* and *∆gnat* mutants in mice, but severe loss of intracellular growth in *ex vivo* macrophages [14] (and this study). Paradoxically, *L*. *major ∆gnd* parasites also display reduced intracellular growth *in vitro* infected macrophages, suggesting that amino sugars remain an important carbon source even in cultured macrophages [13]. Serum that supports growth of cultured macrophages does contain some levels of hyaluronan. It is thus possible that the levels of GlcN/GlcNAc in the phagolysosome of cultured macrophages increase over time, but which is too slow to rescue the highly sensitive ∆*gnat* and ∆*gfat* mutants. Together, our results highlight potentially important differences in the nutrient environment of cultured versus lesion macrophages.

*Leishmania* lack detectable hyaluronidase activity and are unable to breakdown and utilize hyaluronan as a carbon source directly. However, *Leishmania* promastigotes secrete a chitinase and can utilize chitin (a homopolymer of GlcNAc) as their sole carbon source *in vitro*. Overexpression of the *L*. *donovani* chitinase resulted in enhanced lesion development [20], suggesting that this enzyme has a role in virulence. As chitin is not produced by mammals and supplementation of *in vitro* infected macrophages with chitin failed to restore intracellular growth of ∆*gnat*, it is possible that the secreted parasite chitinase complements other hydrolases activities in the macrophage phagolysosome involved in the complete break down of hyaluronan. This would potentially enhance the rate of production of free GlcNAc that can then be used by intracellular amastigotes. Hyaluronan degradation in the phagolysosome will also generate free pools of the acidic sugar, glucuronic acid. *Leishmania* lack enzymes needed to catabolize glucuronic acid and are thus unable to utilize this sugar as sole carbon source (S7 Fig). However, they do contain enzymes needed to convert glucuronic acid to ascorbic acid, an essential vitamin and cofactor for several peroxidases, and this hybrid pathway of *de novo* synthesis may be important for virulence [26,27].

Intriguingly, the saliva of the sandfly vector contains high levels of hyaluronidases, which are injected into the skin together with infective promastigote stages during a natural infection [28,29]. The presence of hyaluronidase in the sandfly saliva correlates with enhanced *L*. *major* induced skin lesion development in susceptible mice [28]. The sandfly hyaluronidase could potentially enhance promastigotes infectivity in a number of ways; by reducing the barrier properties of the extracellular matrix, stimulating neutrophil recruitment and/or angiogenesis via the generation of bioactive low molecular hyaluronan fragments or by generating hyaluronan fragments that are more readily taken up by *Leishmania*-infected macrophages. Secretion of sand fly hyaluronidase is also expected to interfere with the wound healing response that occurs after sand fly bites (and needle puncture), as it depends on an orderly regulation of hyaluronan catabolism [19].

There are a number of reports that other pathogens may utilize hyaluronan as a carbon source, including Group A Streptococcus and *Mycobacteria tuberculosis* [30,31]. In contrast to *Leishmania*, these bacteria secrete hyaluronidases and are therefore able to degrade and use extracellular pools of hyaluronan. In the case of *M*. *tuberculosis*, hyaluronan utilization appears to be important during extracellular growth in alveolar spaces, while intracellular stages are dependent on non-carbohydrate carbon sources [31,32]. Very few microbial pathogens are able to survive and proliferate long term in the mature phagolysosome of macrophages [9,33]. We propose that the dependence of *Leishmania* parasites on amino sugar metabolism, as well as their requirement for many other nutrients [10], may have provided the evolutionary drive to colonize this nutritionally diverse niche in the mammalian host.

## Materials and Methods

### Ethics statement

Use of mice was approved by the Institutional Animal Care and Use Committee of the University of Melbourne (ethics number 0811011.1). All animal experiments were performed in accordance with the Australian National Health Medical Research council (Australian code of practice for the care and use of animals for scientific purposes, 7^th^ Edition, 2004, ISBN: 1864962658).

### Parasite culture

Wild type promastigotes of *L*. *major* (MHOM/SU/73/5ASKH) and *L*. *major* Δ*gnd* were cultured in M199 media (Gibco) supplemented with 10% heat-inactivated foetal bovine serum (FBS) (Gibco) or in completely defined media [34] at 27°C. For isolation of transfected parasites, media were supplemented with bleomycin (5 μg/ml; Calbiochem), nourseothricin (70 μg/ml; Sigma) and G418 (100 μg/ml, Calbiochem) and colonies isolated from M199-agar plates (1%, Nu Sieve agarose, FMC BioProducts). For the isolation of *L*. *major* Δ*gnat* cell line (S1 Text), parasites were additionally supplemented with 50 μg/ml GlcNAc. Metacyclogenesis was determined using peanut agglutinin as described previously [13].

### Metabolic labelling and Western blot analysis of *L*. *major* promastigotes

For labeling of *L*. *major* wild type and Δ*gnat* parasites with [^3^H]-Glc (1 mCi; Perkin Elmer), mid log phase promastigotes (10^8^) were washed twice in PBS and resuspended in M199 media (10^7^ cell/ml) and incubated for 24 hours at 27°C prior to labeling (GlcNAc starve). Parasites were washed once in PBS and incubated in glucose-free RPMI (2 × 10^8^ cell/ml) for 10 minutes at 27°C (Glc starve) before the addition of [^3^H]-Glc (50 μCi/ml) and incubated for a further 30 minutes at 27°C. Thereafter, parasites were resuspended in PBS and extracted in 300 μl chloroform:methanol:water (final ratio of 1:2:0.8 v/v). The extract was partitioned in 1-butanol and water (2:1 v/v) and analyzed by high performance thin layer chromatography (HPTLC) [35]. The de-lipidated pellets were analyzed by 12% SDS-PAGE and Western blotting. After transfer onto nitrocellulose membranes (0.45 μm, Advantec MFS; 100 V, 90 minutes), membranes were incubated in blocking buffer (5% powdered skim milk in 20 mM Tris-HCl, pH 7.6, 300 mM NaCl, and 0.05% Tween 20) overnight at 4°C. Membranes were probed with anti-gp63 (provided by Dr. E. Handman; 1:1000 dilution) and anti-SMP1 rabbit antibodies (1:1000 dilution), or the monoclonal anti-phosphoglycan LT15 (provided by Dr. T. Ilg; 1:1000 dilution), all suspended in blocking buffer for 1 hour at RT. Blots were washed in Tris buffer saline containing Tween-20 (TTBS; 20 mM Tris-HCl, pH 7.6, 300 mM NaCl, 0.05% Tween 20) for 30 minutes. Secondary antibody (horseradish peroxidase-conjugated anti-rabbit and anti-mouse secondary antibodies) were diluted 1:2500 in blocking buffer and applied to blots (1 hour, RT). After washing in TTBS, binding was detected using ECL reagents (Amersham) and analyzed using Gel Pro Analyzer.

### *In vitro* GPI and LLO biosynthesis

Log phase *L*. *major* wild type and Δ*gnat* promastigotes were washed three times in PBS and resuspended in completely defined medium (CDM) (- hexose,—inositol) supplemented with 13 mM glucose, +/- 50 μg/ml GlcNAc for 0, 15, 30, 60 and 120 minutes. Promastigotes were hypotonically lysed (1 mM NaHEPES pH 7.4, 2 mM EGTA, 2 mM DTT, 40 μl/ml protease inhibitor cocktail [PIC] from Roche Diagnostics) by chilling on ice for 10 minutes followed by brief sonication. Cell lysates were centrifuged (0°C, 2,300 x g, 5 minutes) and resuspended in assay buffer (50 mM NaHEPES pH 7.4, 50 mM KCl, 5 mM MgCl_2_, 1 mM MnCl_2_, 2 mM EGTA, 2 mM DTT, 1 mM ATP) containing 40 μl/ml PIC. To begin the assay, membranes were incubated with 50 μCi GDP-[^3^H]-Man (0.25 mCi; Perkin Elmer) (total vol. per assay 80 μl) for 10 minutes in a 27°C water bath. The reaction was stopped by addition of 300 μl chloroform:methanol (1:2 v/v) and samples then extracted for 2 hours at RT with sonication every 20 minutes. Following biphasic separation in 1-butanol and water (2:1 v/v), the organic phases were treated with PI-PLC as described in [36]. Samples were then subjected to another round of biphasic separation in 1-butanol and water (2:1 v/v) and the organic phase resuspended in 40% 1-propanol, loaded onto HPTLC plates and developed in chloroform:methanol:NH_4_OAc:NH_3_:water (180:140:9:9:23 v/v) before being exposed to film at -70°C.

### *In vitro* GNAT activity assay

*L*. *major* promastigotes were suspended in hypotonic buffer (1 mM NaHEPES, pH 7.4, 2 mM EGTA, 2 mM DTT, 40 μl/ml PIC) and chilled on ice for 10 minutes before being lysed by sonication (2 × 4 sec). Following lysis, NaHEPES, pH7.4, MgCl_2_ and acetyl-CoA was added to the lysates to make a final concentration of 50 mM NaHEPES, pH7.4, 5 mM MgCl_2_ and 150 μM acetyl-CoA. GNAT activity in the lysate (3 × 10^7^ cell equivalents in 80 μl) was measured by addition of GlcN6P (1 mM) and incubating at 27°C for 1, 10, 30 or 60 minutes. Controls were either incubated with H_2_O for 60 minutes at 27°C or boiled for 5 minutes before addition of GlcN6P. In all cases, the reaction was stopped by addition of chloroform:methanol (1:2 v/v) to make a final concentration of 1:2:0.8 v/v (chloroform:methanol:water). Samples were extracted (2 hours, RT), and subjected to biphasic separation in 1-butanol and water (2:1 v/v) as described in 0. Aqueous fractions containing the hexose phosphates were analyzed by liquid chromatography mass spectrometry on an Agilent 6520 Q-TOF LC/MS Mass Spectrometer coupled to an Agilent 1200 LC system (Agilent, Palo Alto, CA). All data were acquired and reference mass corrected via a dual-spray electrospray ionization (ESI) source. Each scan or data point on the Total Ion Chromatogram (TIC) is an average of 15,000 transients, producing a spectrum every second. Mass spectra were created by averaging the scans across each peak and background subtracted against the first 10 seconds of the TIC. Acquisition was performed using the Agilent Mass Hunter software version B.02.01 and analysis was performed using Mass Hunter version B.03.01. Mass Spectrometer Conditions: Ionisation mode: Electrospray Ionisation; Drying gas flow: 7 liters/minutes; Nebuliser: 35 psi; Drying gas temperature: 325°C; Capillary Voltage (Vcap): 4000 V; Fragmentor: 100 V; Skimmer: 65 V; OCT RFV: 750 V; Scan range acquired: 100–3000 m/z; Internal Reference ions: Negative Ion Mode = m/z = 112.98 and 1033.98.

### Infection of BALB/c bone marrow-derived and RAW 264.7 macrophages

For the isolation of bone-marrow derived macrophages (BMDMs), tibia and femur of BALB/c mice were flushed with RPMI medium 1640 (Gibco BRL) supplemented with 15% FBS, 4 mM glutamine (MultiCel), 100 units/ml penicillin, 100 μg/ml streptomycin, and 20% (v/v) L929 cell-conditioned media and grown in petri dishes (24 hours, 37°C with 5% CO_2_). BMDMs and RAW 264.7 macrophages were grown on 10 mm coverslips in RPMI medium 1640 (Gibco BRL) supplemented with 15% FBS, 10% L929 conditioned media, 100 units/ml penicillin and 100 μg/ml streptomycin for 24 hours at 37°C with 5% CO_2_. Macrophage monolayers were overlaid with 4-day stationary phase *L*. *major* promastigotes (parasites added to macrophages at a ratio of 10:1) and incubated for 4 hours at 33°C with 5% CO_2_. Coverslips were washed three times in PBS to remove unattached parasites then incubated in fresh macrophage medium (RPMI + 15% FBS) for up to 6 days at 33°C in 5% CO_2_. Chitin (crab shells, Sigma), high molecular weight hyaluronan (Streptococcus isolate, Sigma) or polymers with defined molecular weight (obtained after acid hydrolysis and high performance liquid chromatography and size exclusion chromatography and multiple-angle laser light scattering [HPLC SEC-MALLS]) were added to macrophage cultures at 10 μg/ml. Coverslips were washed in PBS to remove unattached parasites and sequentially incubated in methanol (25°C, 10 minutes), PBS containing 50 mM NH_4_Cl (25°C, 10 minutes), and 1% BSA in PBS (25°C, 30 minutes). The fixed cells were probed with mAb anti-LAMP (1:100 dilution in 1% BSA in PBS; BD Biosciences) and Alexa Fluor-488 goat anti-rat (1:1000 dilution; Molecular Probes) to visualize PV membranes. Infected macrophages were incubated with 0.2 μg/ml propidium iodide (Sigma) and mounted in Mowiol 4–88 (5 μl; Calbiochem) containing Hoechst 33342 (8 μg/ml; Life Technologies) to visualize the plasma membrane, nuclei and kinetoplasts, respectively. Hyaluronan was detected by live-cell microscopy by covalently linking high molecular weight polymers to fluorescein (FITC) [37]**.** Macrophages were infected with *L*. *major* or *L*. *mexicana* (M379) for 24 hours before adding hyaluronan-FITC for an additional 18 hours and LysoTracker-Red DND-99 (Invitrogen) for 60 minutes. Fluorescence microscopy was performed using a Zeiss Axioplan2 imaging microscope, equipped with AxiCam MRm camera and the AXIOVISION 4.3 software (Zeiss). Images were compiled in Photoshop Elements.

### Mice infections

Female BALB/c mice (6–8 weeks old) were maintained in a pathogen-free facility (Bio21 Institute, University of Melbourne). Groups of mice (five per treatment) were inoculated intradermally at the base of the tail with stationary-phase promastigotes (10^6^ in 50μl sterile PBS) or lesion-derived amastigotes (2 × 10^5^ parasites in 50 μl sterile PBS). The size of the developing lesions was monitored and recorded weekly as described in [38]. Data is expressed as arithmetic mean (± standard error mean) of the lesion scores for the groups of five mice. For amastigote infections, excised lesions (5–10 mm) from BALB/c mice were placed in chilled 1 × PBS and pushed through a plastic sieve using the flat end of a 5 ml syringe plunger and syringed three times using a 27 ½ gauge needle. Amastigotes were separated from cell debris by centrifugation (30 x g, 10 minutes, 4°C) and the pellet washed twice in chilled PBS (850 x g, 10 minutes, 4°C). Amastigote numbers were determined using a haemocytometer.

### Analysis of hyaluronan turn over in skin lesions

*L*. *mexicana* infected BALB/c mice containing skin lesions of score of 2 were given an intraperitoneal bolus of 35 μl/g bodyweight ^2^H_2_O (Cambridge Isotope Laboratories) and 0.9% NaCl and subsequently fed with 9% ^2^H_2_O in their drinking water for up to 5 weeks. This resulted in the stable enrichment of 5% ^2^H_2_O of total body water as determined by [39]. Excised, lesions were cut into small fragments (about 1mm^3^) and digested with hyaluronidase (Type I; Sigma) for 18 hours at 37°C. After biphasic extraction with chloroform and methanol (1:2 v/v), the released tetrasaccharides in the aqueous phase were analyzed by liquid chromatography and mass spectrometry (LC-MS) using an LC-QTOF-MS—an Agilent 1290 LC-system coupled to an Agilent 6550 Electrospray Ionisation-Quadrupole Time of Flight (Agilent Technologies, Singapore). Tandem MS was used to distinguish hyaluronic acid (HA) from N-acetylheparosan [40]. Amino sugar species were separated by injecting 10 μL of samples into Phenomenex Hyperclone C18 column –3.8 μm 4.6 x 100 mm with a flow rate of 200 μL/minutes. The mobile phases used were 10 mM ammonium acetate (Sigma Aldrich) in water (A) and then 10 mM ammonium acetate in acetonitrile (B). The column temperature was kept at 20°C with a 10 minute run time. The chromatographic separation of the amino sugars was achieved by a gradient of mobile phase B increased from 1% to 50% during the first 6 minutes, held for two minutes followed by equilibrating the column for another two minutes as the initial composition. Mass spectra for the ions were collected in negative ionization mode. The full scan spectra (100–1700 Da) was collected using the following conditions as fragmentor: voltage 175 V, nebulizer pressure 45 psi and capillary voltage 4000 V. Nitrogen was used as a drying (13 L/minutes) and sheath gas flow as 12 L/minutes with sheath gas temperature 275°C. Total ion chromatograms (TIC) and mass spectra were processed using an Agilent MassHunter software, version 6. The ion 775.2 was chosen as a diagnostic ion following the analysis of digested HA standards. The M_0_, M_1_, M_2_, M_3_, and M_4_ were quantitated. The half-life was determined by plotting the fraction of new HA (excess molar enrichment divided by the maximal excess molar enrichment (4 weeks labeled lesion)) over time.

## Supporting Information

S1 Fig GNAT sequence alignment. GNAT sequences from *Homo sapiens* (NP_932332), *Caenorhabditis elegans* (NP_505654), *Saccharomyces cerevisiae* (YFL017C), *Trypanosoma brucei* (Tb11.01.2886), *Trypanosoma cruzi* (Tc00.1047053511671.70) and *Leishmania major* (LmjF38.005) were aligned using T-COFFEE and edited with BOXSHADE. Conserved residues that are identical or similar are boxed in black and grey, respectively. Residues marked with an asterisk (Ile97, Glu98, Asp99, Val102, Gly112, Leu115, Ile116, Phe142, Tyr143 and Gly147) indicate highly conserved amino acids involved in binding and proper positioning of substrate (GlcN6P) and/or co-factor (acetyl-CoA) as determined by studies performed in *S*. *cerevisiae*. Grey arrow denotes the highly conserved Tyr143, which is essential for catalysis.
(TIF)

S2 Fig Deletion of *GNAT* in *L*. *major*. (A) ∆*gnat* parasites were generated by homologues recombination using bleomycin and nourseothrecin resistant cassettes (S1 Text). The primers used to verify the integration of resistant cassette and loss of GNAT by PCR are indicated. (B) WT and ∆*gnat* genomic DNA was used as a template in PCR with primers as follows: lane 1–1 and 4; Lane 2–1 and 2 (specific for bleomycin marker); Lane 3–1 and 5 (specific for SAT marker); Lane 4–3 and 4. (C) WT and ∆*gnat* promastigotes were cultured in media with or without GlcNAc (50μg/ml) at 27°C for 72 hours and cell morphology and viability was assessed by differential contract images.
(TIF)

S3 Fig Lesion derived ∆*gnat* express glycolipids in the absence of *GNAT*. (A) The glycolipids of lesion-extracted amastigotes (EA) of WT and ∆*gnat* parasites were analysed by HPTLC and orcinol-stained. WT promastigotes (Pro) were used as a control, showing the major free GPIs, GIPL1, 2 and 3. (B) PCR confirmed loss of *GNAT* in lesion-derived amastigotes. Primers for each lane were used as described in S2B Fig whereby lane 2 and 3 indicate retention of SAT and BLEO resistance cassettes and lane 1 and 4 loss of *GNAT* in ∆*gnat*, but not WT amastigotes. (C) Growth of lesion derived WT and ∆*gnat* promastigotes in media with or without GlcNAc (50 μg/ml) as determined by optical density at 600 nm at day 4.
(TIF)

S4 Fig Hyaluronan is turned over in *Leishmania* induced skin lesions. Mice containing *L*. *mexicana* induced skin lesions were labeled with 5% ^2^H_2_O, lesions were excised and deuterium enrichment of stable C-H bonds of newly synthesized hyaluronan (HA) was determined by LC-MS detection of the diagnostic tetrasaccharide (m/z 775.2) (depicted in top right panel) after 1, 2, 4 or 5 days or 4 weeks. The relative abundance of the tetrasaccharide isotopomers M_0_, M_1_, M_2_, M_3_, and M_4_ are shown on the left panel and M_2_ quantification on the right panel. Data represent mean and SD from three separate experiments.
(TIF)

S5 Fig *L*. *major*-induced phagolysosomes accumulate FITC-conjugated hyaluronan. (A) and (B) RAW 264.7 macrophages were infected with *L*. *major* expressing cytosolic mCherry for 24 hours and then incubated with FITC-conjugated hyaluronan (HA::FITC). Live-macrophages were analyzed by fluorescence microscopy. Scale bar = 10μm.
(TIF)

S6 Fig ∆*gnat* amastigotes induce delayed skin lesions in susceptible mice. BALB/c mice were infected intradermally with lesion-derived WT and ∆*gnat* amastigotes (10^5^) and lesion progression was scored weekly. Mean and SEM shown (n = 5 mice).
(TIF)

S7 Fig *L*. *major* promastigotes are unable to utilize glucuronic acid as major carbon source. *L*. *major* promastigotes were cultured in completely defined media [13] containing either glucose (10 mM), glucuronic acid (10mM) or no hexose and growth was detected by optical density at 600nm over time.
(TIF)

S1 Text Experimental procedures and references. (DOCX)
