# Supplementary figures and images for: Intracellular Survival of Leishmania major Depends on Uptake and Degradation of Extracellular Matrix Glycosaminoglycans by Macrophages
Source: PLoS Pathog. 2015 Sep 3;11(9):e1005136. doi: 10.1371/journal.ppat.1005136 (PMC4559419; doi:10.1371/journal.ppat.1005136)

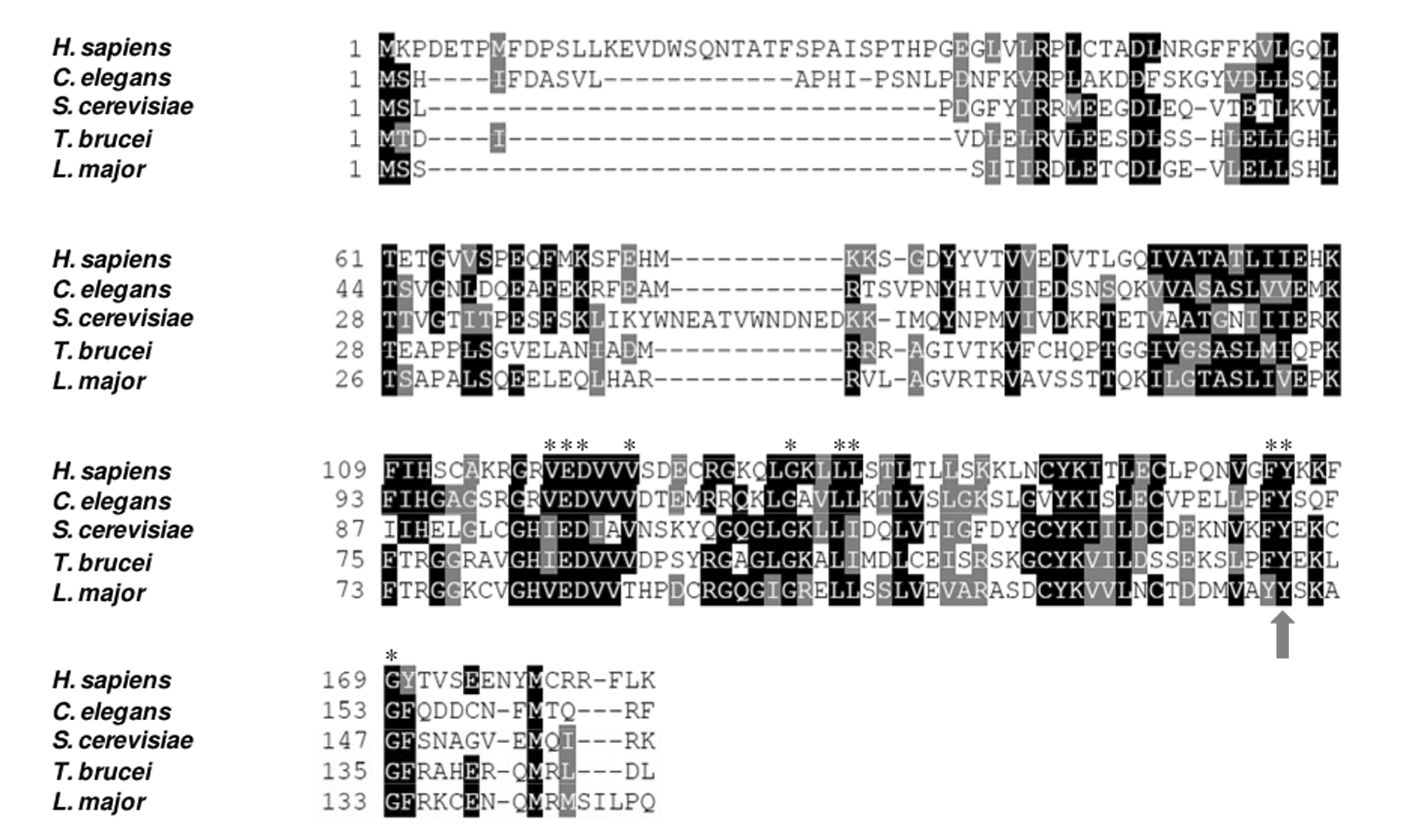

Supplement: S1 Fig — GNAT sequences from Homo sapiens (NP_932332), Caenorhabditis elegans (NP_505654), Saccharomyces cerevisiae (YFL017C), Trypanosoma brucei (Tb11.01.2886), Trypanosoma cruzi (Tc00.1047053511671.70) and Leishmania major (LmjF38.005) were aligned using T-COFFEE and edited with BOXSHADE. Conserved residues that are identical or similar are boxed in black and grey, respectively. Residues marked with an asterisk (Ile97, Glu98, Asp99, Val102, Gly112, Leu115, Ile116, Phe142, Tyr143 and Gly147) indicate highly conserved amino acids involved in binding and proper positioning of substrate (GlcN6P) and/or co-factor (acetyl-CoA) as determined by studies performed in S. cerevisiae. Grey arrow denotes the highly conserved Tyr143, which is essential for catalysis. (TIF) [file ppat.1005136.s001.tif]

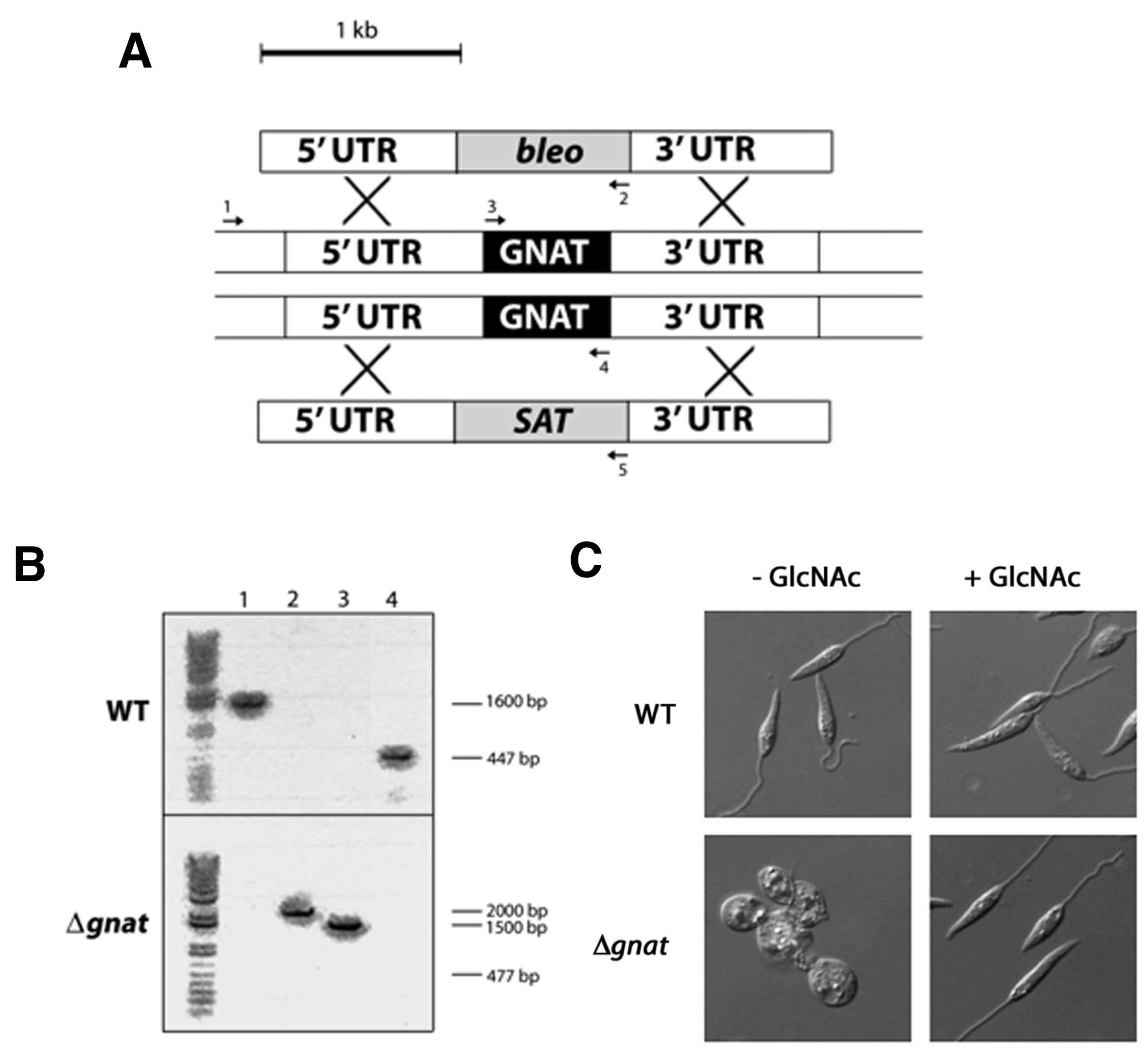

Supplement: S2 Fig — (A) ∆gnat parasites were generated by homologues recombination using bleomycin and nourseothrecin resistant cassettes (S1 Text). The primers used to verify the integration of resistant cassette and loss of GNAT by PCR are indicated. (B) WT and ∆gnat genomic DNA was used as a template in PCR with primers as follows: lane 1–1 and 4; Lane 2–1 and 2 (specific for bleomycin marker); Lane 3–1 and 5 (specific for SAT marker); Lane 4–3 and 4. (C) WT and ∆gnat promastigotes were cultured in media with or without GlcNAc (50μg/ml) at 27°C for 72 hours and cell morphology and viability was assessed by differential contract images. (TIF) [file ppat.1005136.s002.TIF]

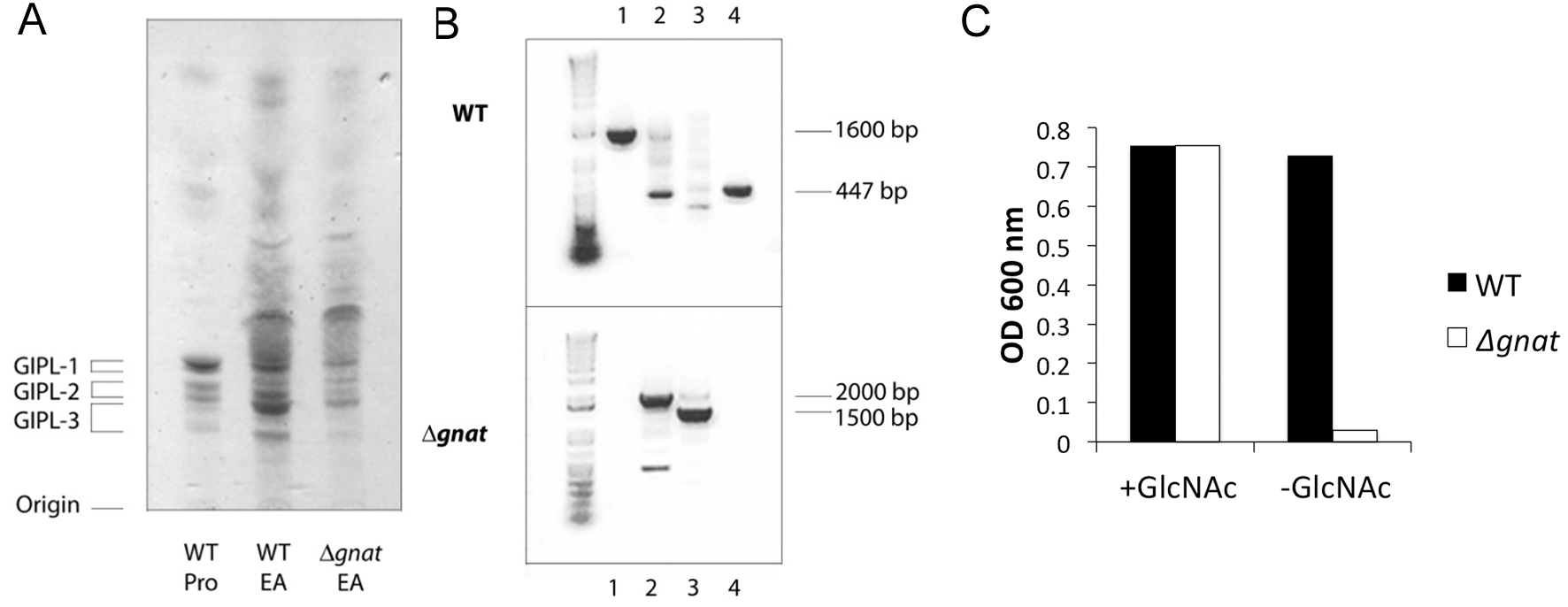

Supplement: S3 Fig — (A) The glycolipids of lesion-extracted amastigotes (EA) of WT and ∆gnat parasites were analysed by HPTLC and orcinol-stained. WT promastigotes (Pro) were used as a control, showing the major free GPIs, GIPL1, 2 and 3. (B) PCR confirmed loss of GNAT in lesion-derived amastigotes. Primers for each lane were used as described in S2B Fig whereby lane 2 and 3 indicate retention of SAT and BLEO resistance cassettes and lane 1 and 4 loss of GNAT in ∆gnat, but not WT amastigotes. (C) Growth of lesion derived WT and ∆gnat promastigotes in media with or without GlcNAc (50 μg/ml) as determined by optical density at 600 nm at day 4. (TIF) [file ppat.1005136.s003.TIF]

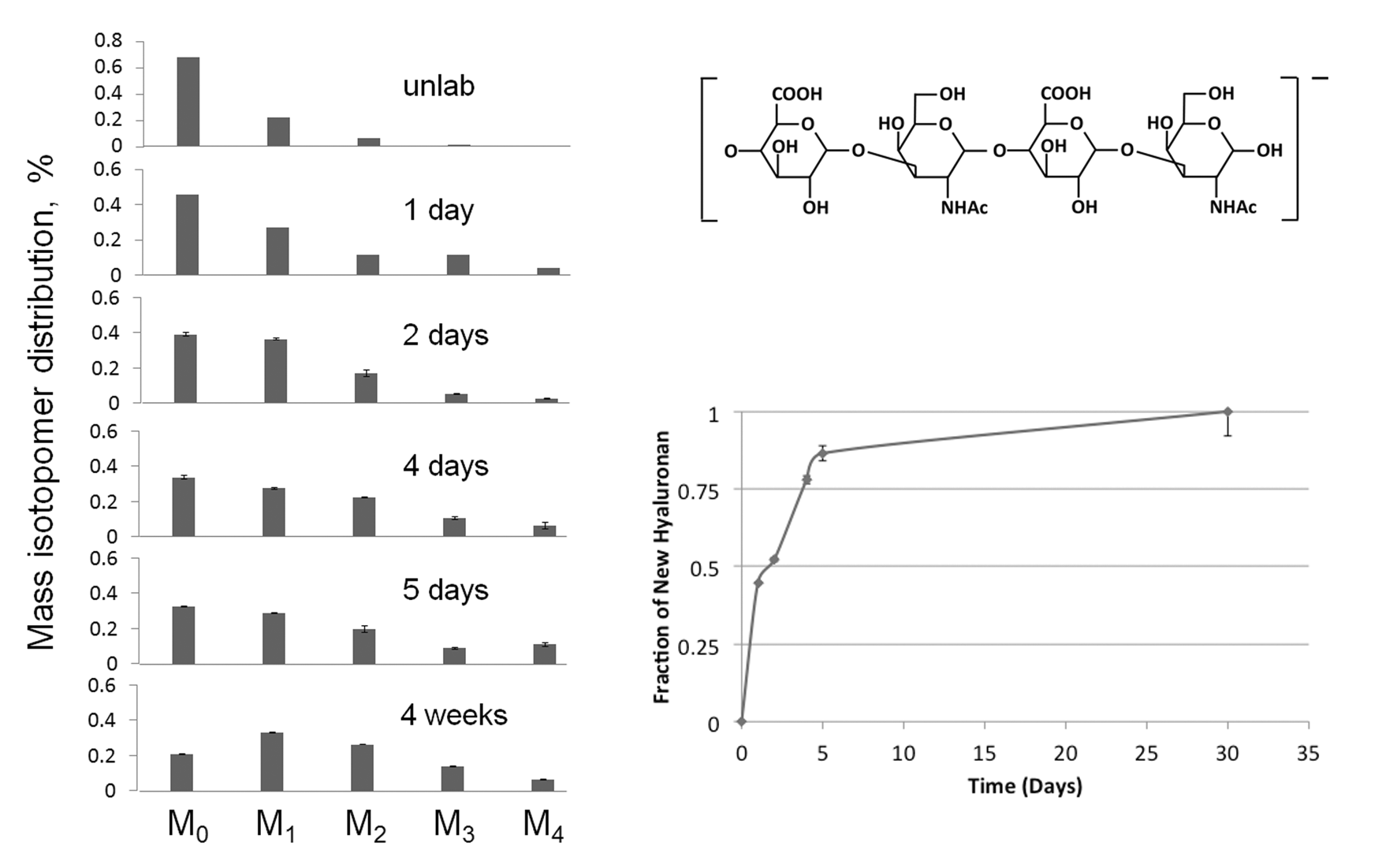

Supplement: S4 Fig — Mice containing L. mexicana induced skin lesions were labeled with 5% 2H2O, lesions were excised and deuterium enrichment of stable C-H bonds of newly synthesized hyaluronan (HA) was determined by LC-MS detection of the diagnostic tetrasaccharide (m/z 775.2) (depicted in top right panel) after 1, 2, 4 or 5 days or 4 weeks. The relative abundance of the tetrasaccharide isotopomers M0, M1, M2, M3, and M4 are shown on the left panel and M2 quantification on the right panel. Data represent mean and SD from three separate experiments. (TIF) [file ppat.1005136.s004.tif]

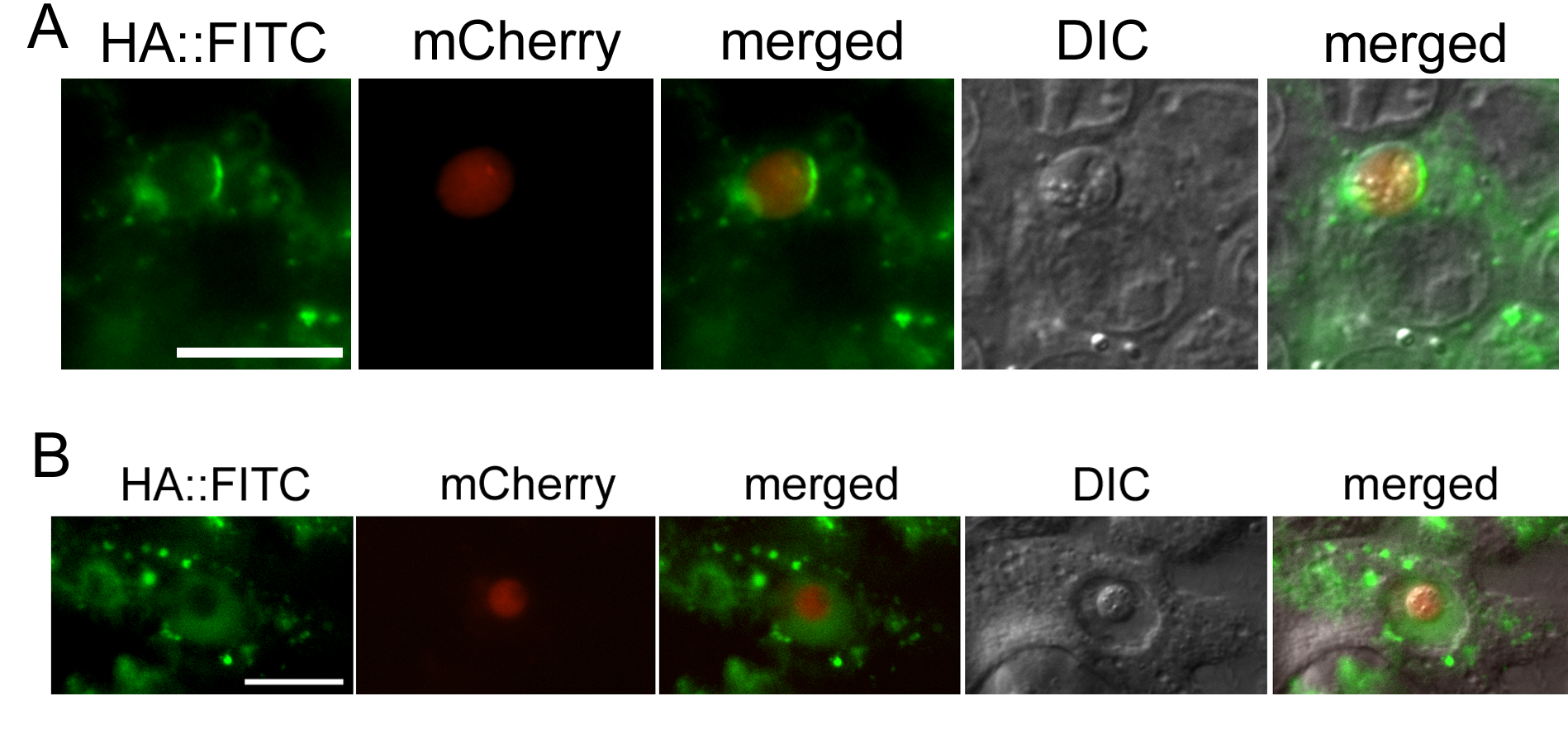

Supplement: S5 Fig — (A) and (B) RAW 264.7 macrophages were infected with L. major expressing cytosolic mCherry for 24 hours and then incubated with FITC-conjugated hyaluronan (HA::FITC). Live-macrophages were analyzed by fluorescence microscopy. Scale bar = 10μm. (TIF) [file ppat.1005136.s005.tif]

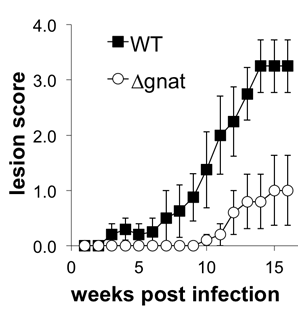

Supplement: S6 Fig — BALB/c mice were infected intradermally with lesion-derived WT and ∆gnat amastigotes (105) and lesion progression was scored weekly. Mean and SEM shown (n = 5 mice). (TIF) [file ppat.1005136.s006.TIF]

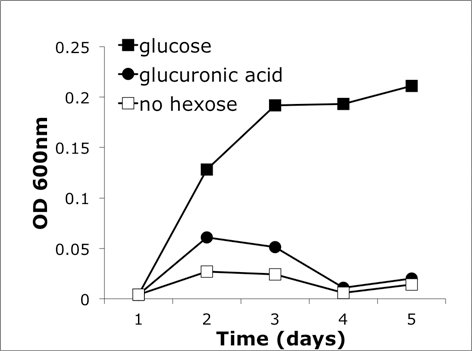

Supplement: S7 Fig — L. major promastigotes were cultured in completely defined media [13] containing either glucose (10 mM), glucuronic acid (10mM) or no hexose and growth was detected by optical density at 600nm over time. (TIF) [file ppat.1005136.s007.TIF]
