# Supplementary material for: Intracellular Survival of Leishmania major Depends on Uptake and Degradation of Extracellular Matrix Glycosaminoglycans by Macrophages
Source: PLoS Pathog. 2015 Sep 3;11(9):e1005136. doi: 10.1371/journal.ppat.1005136 (PMC4559419; doi:10.1371/journal.ppat.1005136)
Supplement: S1 Text — (DOCX) [file ppat.1005136.s008.docx]

**Experimental Procedures**

## *Generation of GNAT and GND gene-replacement cassettes*

The replacement cassette for targeted gene deletion of *L. major* GNAT was generated by using genomic DNA isolated from *L. major* wild type promastigotes as a template in a polymerase chain reaction (PCR). The 5’ untranslated region (5’UTR) and 3’UTR of GNAT were amplified using the primers GGCAAGCTTTCGGCATCGACGCCAGGGTT and GGCGAATTCTGGCCGCACGGGTGAAGGTT for 5’UTR of GNAT; GGCGGATCCTCGACCTCGGAGTGTACCTGA and GGCTCTAGATTCAGGTAGTCACCAAGATC for 3’UTR of GNAT.

The HindIII/EcoRI-cut 5’UTR fragment was cloned into the HindIII and EcoRI sites of pBluescript II SK (Stratagene), followed by ligation of the BamHI/XbaI-cut 3’UTR fragment into the *Bam*HI and XbaI sites of the pBluescript vector containing the 5’UTR. The bleomycin and nourseothricin resistant cassettes were obtained from pXG-BLEO and pXG-SAT, respectively, by digesting with XhoI, followed by blunt-end treatment using Klenow polymerase (New England BioLabs) following digestion. After heat inactivation of Klenow polymerase, plasmids were then digested using BamHI. The resistant cassettes were isolated by gel purification (Qiagen gel purification kit) and cloned between the 5’ and 3’UTR using the SmaI and BamHI sites of the pBluescript vector containing the 5’ and 3’ UTRs. The complete knockout constructs were verified by diagnostic digests and DNA sequencing. The BLE- and SAT-containing GNAT gene replacement cassettes were excised from the plasmid by HindIII/XbaI digestion, gel-purified and the DNA (5 μg) transfected into *L. major* promastigotes. For the complemention, the complete open reading frame of GNAT was PCR amplified using primers GGCGGATCCATGAGTAGCTCTATCATCATC and GGCGAATTCTCATTGGGGTAAAATGCTCA and cloned into BamHI and EcorI sites of pX-PAC. All constructs were verified by diagnostic digests and DNA sequencing (Department of Pathology, University of Melbourne). *Leishmania* transfections and isolation of clonal cell lines by agarose-plating were performed as described before [1].

**Supporting reference list**

1. Robinson KA, Beverley SM (2003) Improvements in transfection efficiency and tests of RNA interference (RNAi) approaches in the protozoan parasite Leishmania. Mol Biochem Parasitol 128: 217-228.
